# Non-Equilibrium Molecular Dynamics Simulation Captures Potential New Drug Target: Ec-MscL Gating Mechanism Explored with External Electric Field

**DOI:** 10.3390/antibiotics15090923

**Published:** 2026-09-18

**Authors:** Fengyang Han, Robin Wray, Paul Blount, Junmei Wang

**Affiliations:** 1 Department of Pharmaceutical Sciences and Computational Chemical Genomics Screening Center, School of Pharmacy, University of Pittsburgh, Pittsburgh, PA 15261, USA; feh34@pitt.edu; 2 Department of Physiology, UT Southwestern Medical Center, 5323 Harry Hines Blvd, Dallas, TX 75390, USA; robin.wray@utsouthwestern.edu

**Keywords:** mechanosensitive ion channels, Ec-MscL, channel gating dynamics, mechanosensitive channel targeting, antibiotic discovery

## Abstract

**Background:** The emergence of multidrug-resistant bacteria, coupled with the stagnation of antibiotic discovery, underscores the urgent need for new therapeutic strategies targeting underexplored bacterial mechanisms. The *Escherichia coli* mechanosensitive channel of large conductance (Ec-MscL) is a highly conserved bacterial membrane protein that regulates osmotic homeostasis through gated pore formation; however, its fully open conformation remains structurally unresolved due to experimental limitations. Building upon our previous studies, in which an external electric field (EEF) was used to accelerate the translocation of the antibiotic dihydrostreptomycin (DHS) through Ec-MscL, we extend this approach to investigate peptide-scale gating transitions and identify the partially open conformation of this channel for subsequent binder design. **Methods:** In this work, we employed EEF-steered molecular dynamics (MD) simulations to model the translocation of the experimentally validated permeant bovine pancreatic trypsin inhibitor (BPTI), a small positively charged peptide, through Ec-MscL. This approach enabled the capture and stabilization of an a partially open-channel conformation of Ec-MscL, which was subsequently evaluated using QMEAN structural quality assessment and MM/PBSA free-energy calculations. Using this open-state structure as a template, we performed virtual screening against a curated antibiotic library from the Community for Open Antimicrobial Drug Discovery (CO-ADD), followed by conventional MD simulations of the top-ranked candidates. **Results:** Eight ligands were computationally identified that stably interact with the channel and were predicted to inhibit its closure, highlighting their potential as antibiotics or antibiotic adjuvants. In addition, four commercially available compounds were evaluated in cell viability assays, with the results suggesting that compound abJ0y affects bacterial growth. **Conclusions:** Together, our study establishes a structure-guided workflow for identifying functional states of mechanosensitive channels and presents a computational pipeline for antibiotic discovery, which was further supported by the identification of a compound that reduced growth in MscL-expressing cells with potential antibacterial activity.

## 1. Introduction

Mechanosensitive (MS) channels are valve-like membrane proteins that maintain cellular integrity by relieving osmotic pressure through transient pore formation to allow solutes flow in and out [1,2,3]. Among them, the mechanosensitive channel protein of large conductance (MscL) is particularly notable for its structural simplicity, high conservation across bacterial species, and its ability to form large pores (~30 Å) that allow the passage of small solutes and peptides, such as bovine pancreatic trypsin inhibitor (BPTI) [4,5,6]. Due to its essential role in osmoprotection and absence in humans, MscL has been proposed as one of the top 20 potential drug targets [7], making it an attractive target for the development of novel antibacterial therapeutics [8,9,10].

Despite its potential as a therapeutic target, efforts to structurally resolve the fully open conformation of MscL have remained unsuccessful, largely due to the difficulty of applying sustained membrane tension in vitro and the absence of pharmacological tools capable of stabilizing distinct gating states. Molecular dynamics (MD) simulations provide a powerful alternative for probing conformational transitions in membrane proteins; however, the timescales required to capture rare gating events generally exceed those accessible to conventional MD simulations. Several computational studies have attempted to induce opening of the *Mycobacterium tuberculosis* homolog of MscL (Mt-MscL), which shares a conserved architecture with Ec-MscL. Even with 20 μs simulations under a membrane tension of 50 mN/m, the resulting pore opening was limited to approximately 5 Å, close to the structural threshold required to maintain membrane integrity [11]. These findings reinforce the long-standing view that membrane tension alone is often insufficient to fully access the open state of MscL, even on extended simulation timescales. In comparison, *Escherichia coli* MscL (Ec-MscL), which requires lower membrane tension for gating and has been extensively characterized experimentally, has emerged as a preferred model system for both experimental and computational investigations of mechanosensitive channel activation [12].

The limitations of conventional MD simulations have motivated the development and application of non-equilibrium molecular dynamics methods to investigate rare events and externally driven processes. Techniques such as steered MD [13], umbrella sampling [14], Gaussian accelerated MD (GaMD) [15], ultrasound-based simulations [16], and the dynamical nonequilibrium molecular dynamics (D-NEMD) approach [17] have been widely applied to biomolecular systems to probe mechanical responses, transport phenomena, and enhanced conformational sampling.

In the context of Ec-MscL, our previous study employed an external electric field (EEF) to simulate the translocation of dihydrostreptomycin (DHS) through the channel [18]. These simulations identified key hotspots involved in the permeation pathway, as well as ligands capable of blocking DHS passage, both of which were subsequently validated experimentally. The results demonstrated that a directional EEF selectively applied to the ligand can accelerate translocation events in a physically interpretable manner. Importantly, the simulations reproduced experimental observations showing that elevated membrane potential enhances DHS activity. Collectively, these findings established the EEF-based approach as a non-equilibrium yet physically relevant strategy for investigating gating transitions and ligand permeation in Ec-MscL.

In the present study, we extend this strategy beyond small-molecule permeants by applying an external electric field (EEF) to bovine pancreatic trypsin inhibitor (BPTI) [19], a positively charged 58-residue peptide previously shown to permeate MscL in dual-color fluorescence and fluorescence correlation spectroscopy (FCS) experiments [5]. Unlike DHS, the larger size and more complex charge distribution of BPTI impose a substantially greater challenge on the channel gating machinery, thereby providing an opportunity to investigate not only permeation but also the partially open conformation of Ec-MscL. Notably, larger biomolecules such as the histidine-containing phosphocarrier protein HPr (9119 Da) were reported to be incapable of permeating MscL, thereby establishing an approximate upper size limit for channel permeants.

In this study, we developed an integrated computational and experimental framework to identify small-molecule modulators of MscL. We first used electric-field-guided simulations to characterize a metastable open-channel conformation of Ec-MscL, which was subsequently used as a structural model for structure-based virtual screening. Candidate compounds were then evaluated through molecular dynamics simulations and experimental cell-based assays to assess their effects on MscL gating and bacterial viability. This approach identified several compounds that delayed or inhibited MscL channel closure, including one compound that exhibited measurable inhibitory activity against MscL-expressing bacterial strains. Overall, this study establishes an integrated pipeline spanning electric-field-guided conformational sampling to in vitro validation, enabling the rational design of small-molecule modulators targeting MscL [20].

## 2. Results and Discussion

### 2.1. The Passage of BPTI via External Electric Field-Steered Molecular Dynamics Simulation

**Figure 1:**
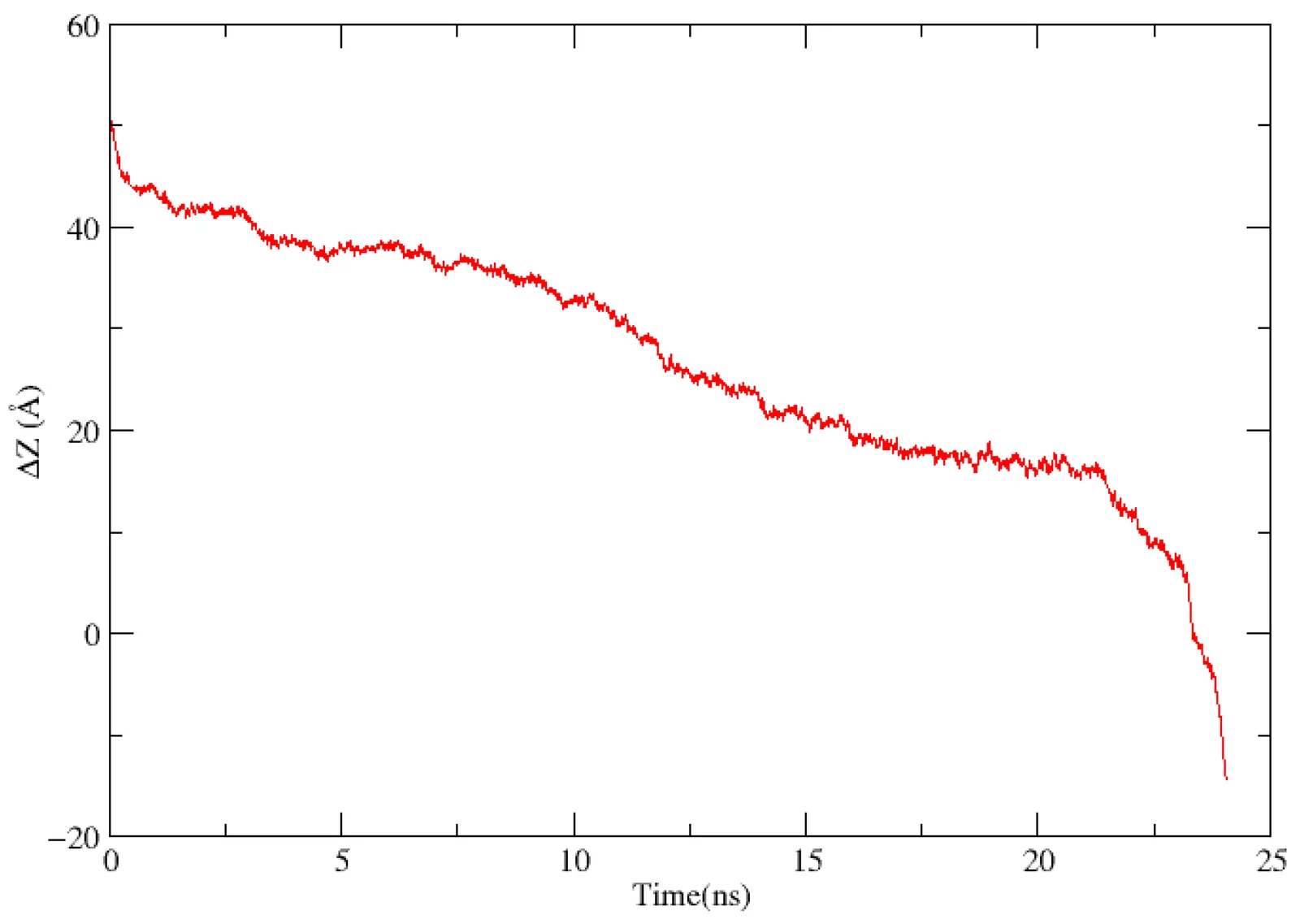
Passage of BPTI during EEF-steered molecular dynamics simulation for BPTI conformation 6 under an external electric field of 0.1 V/Å. ΔZ was defined as the difference in the z-coordinates between the geometric center of BPTI and the geometric center of the five lysine residues (K106, K242, K378, K514, and K650) within the pentameric Ec-MscL channel. The trajectory shows that BPTI successfully translocated through the Ec-MscL channel within approximately 25 ns.

The passage of BPTI through the Ec-MscL channel was monitored by calculating the ΔZ coordinate, defined as the distance along the Z-axis between the centroid of the BPTI molecule and the geometric center of the five bottom lysine residues at the terminus of TM2. Under external electric fields (EEFs) ranging from 0.1 to 0.2 V/Å, successful BPTI translocation events were observed for all six initial BPTI conformations, with passage times ranging from 2 to 80 ns. Notably, for initial BPTI conformation 6, successful passage was achieved even under a lower EEF of 0.1 V/Å (Figure 1). For this system, on average, we can sample 26 nanoseconds using NVIDIA GTX 1080ti. For three EEF 0.10, 0.15, and 0.20 V/Å, the total accumulated production times from all six conformations were 464.00, 105.99, and 70.67 ns, respectively. Movies of three passages for conformation 6 are provided as Supplementary Materials.

**Figure 2:**
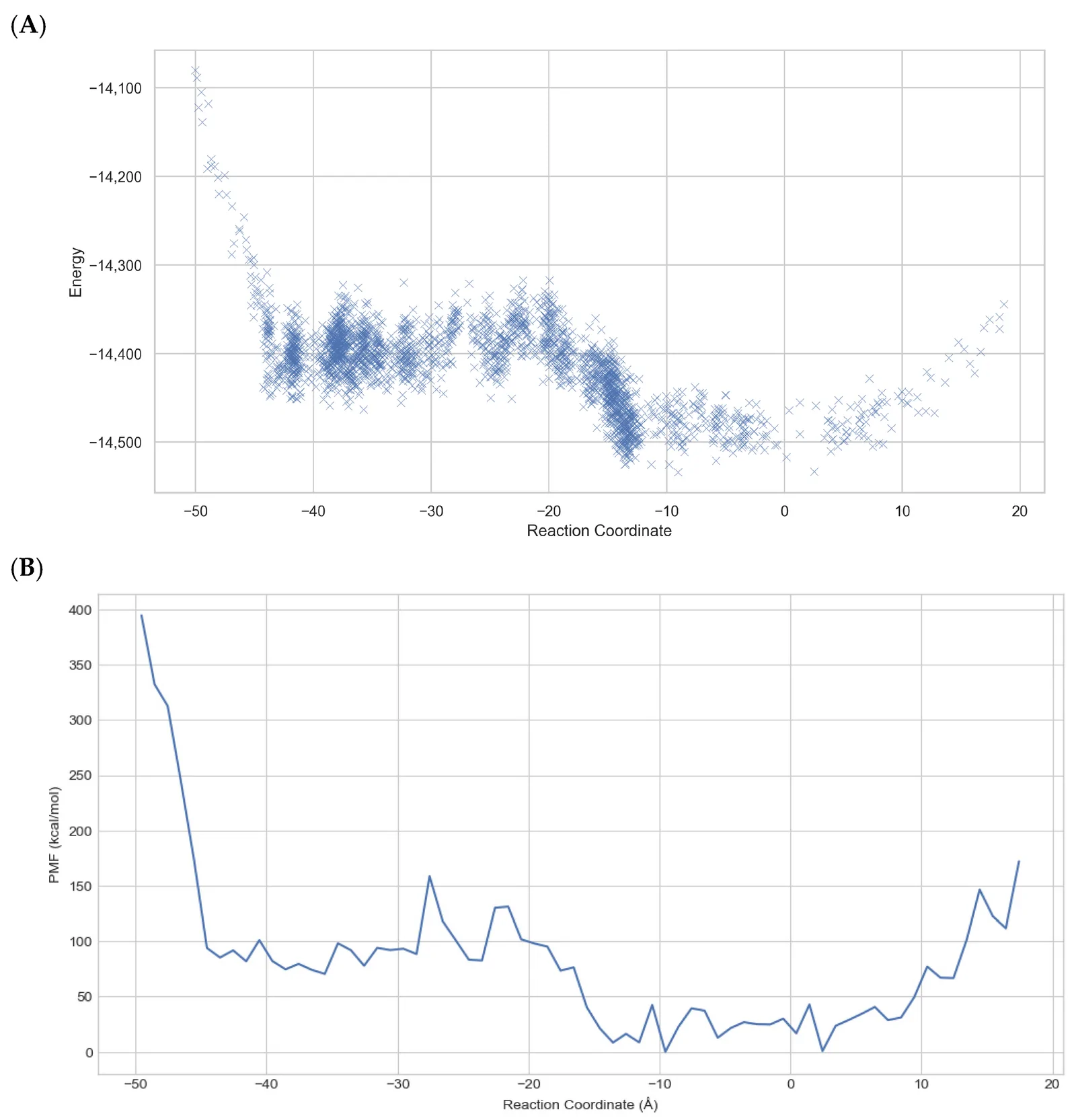
Distribution of MM/PBSA free energies (**A**) and the corresponding MM/PBSA energy profile (**B**) along the reaction coordinate ΔZ.

To investigate the BPTI translocation process under weaker EEFs, we performed extensive MD simulations of the BPTI–MscL system with an accumulated simulation time of approximately 1 μs. However, due to the intrinsically slow dynamics of EEF-driven translocation, no complete passage events were observed under electric fields of 0.01 or 0.05 V/Å within this timescale. Under these weaker EEF conditions, BPTI predominantly became trapped at ΔZ = −35 Å, which was subsequently identified as an energy barrier by MM-PBSA free-energy calculations, appearing as a region with elevated MM-PBSA free energy and MM-PBSA energy profile values (Figure 2). These results suggest that a minimum threshold of EEF strength may be required to drive BPTI translocation through the Ec-MscL channel. They also highlight the challenges of capturing rare events and slow transport processes, even using non-equilibrium MD simulations rather than conventional equilibrium MD simulations.

### 2.2. Quality Assessment of the Open-Channel MscL Conformations

**Figure 3:**
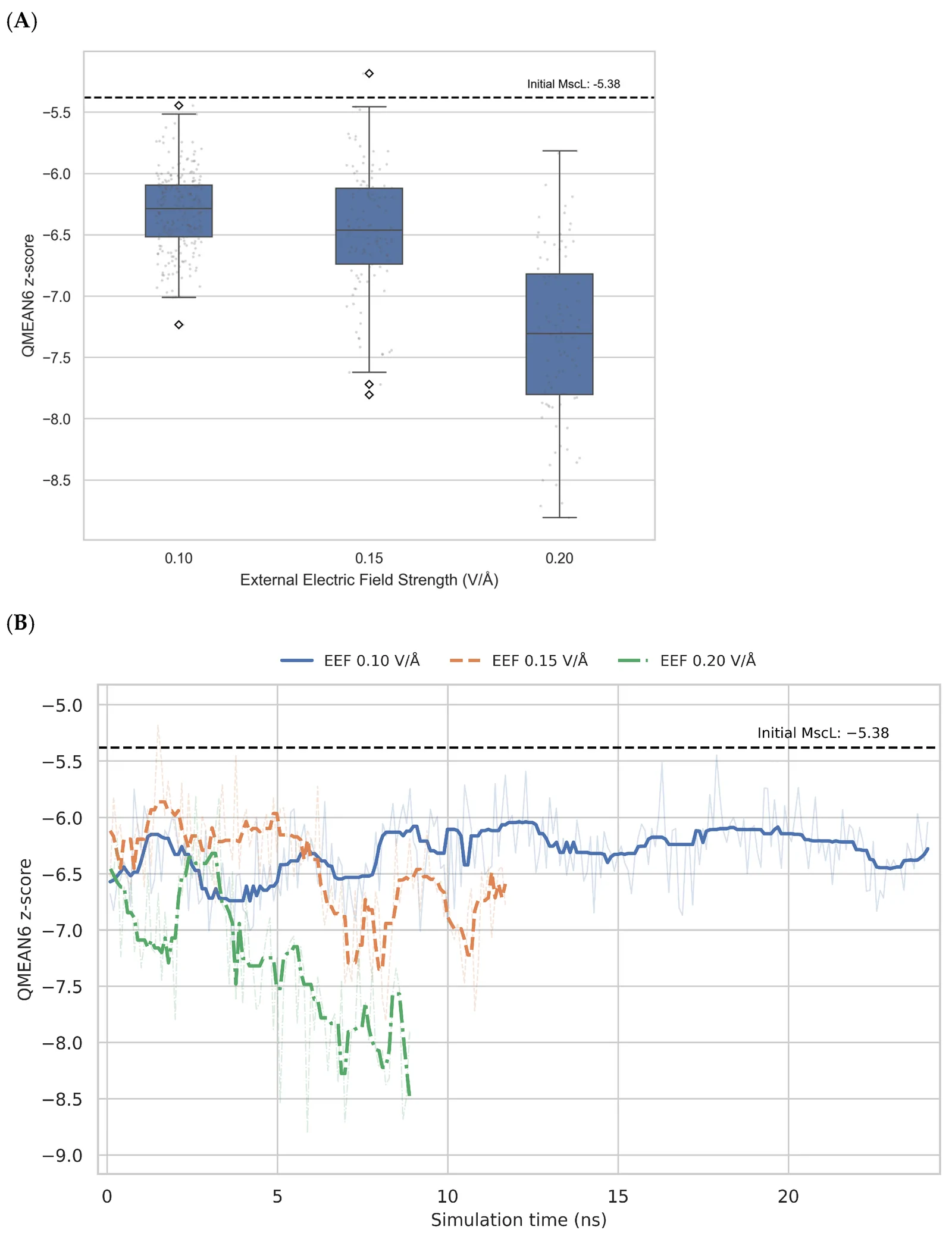
Structural quality assessment of Ec-MscL conformations sampled under different EEF conditions using the QMEAN6 score. (**A**) Distributions of QMEAN6 Z-scores calculated from snapshots sampled from trajectories subjected to EEF strengths of 0.10, 0.15, and 0.20 V/Å. The horizontal fine dashed line indicates the QMEAN6 Z-score of the initial Ec-MscL model (−5.38). Whiskers extend to 1.5 times the interquartile range, and diamonds in the figure indicate observations beyond the whiskers. (**B**) Temporal evolution of QMEAN6 Z-scores along the trajectories under the three EEF conditions. A rolling median was applied to facilitate visualization of temporal trends.

The initial Ec-MscL structure had a QMEAN6 Z-score of −5.38 and was used as a reference for comparison. QMEAN6 Z-scores were calculated for snapshots sampled along the EEF trajectories using the QMEAN server. Figure 3A summarizes the distributions of QMEAN6 Z-scores. The 0.10 and 0.15 V/Å trajectories showed substantial overlap, whereas the 0.20 V/Å trajectory exhibited generally lower and more broadly distributed QMEAN6 scores. Figure 3B shows the temporal evolution of QMEAN6 Z-scores. The 0.10 V/Å trajectory remained comparatively stable over the sampled period, whereas a gradual decrease was observed for the 0.20 V/Å trajectory. Because the 0.10 and 0.15 V/Å trajectories exhibited similar QMEAN6 behavior, the lower-field 0.10 V/Å condition was selected as the more conservative condition for subsequent conformational analysis.

The successful passage of BPTI is consistent with previous experimental evidence showing that activated MscL allows BPTI to pass through the channel [5]. Previous simulations used an external electric field to facilitate the passage of the smaller antibiotic dihydrostreptomycin through MscL [18]. In the present study, we extended this approach to BPTI, using an external electric field to facilitate peptide translocation and to sample expanded channel conformations for subsequent screening.

### 2.3. Structural Clustering and Open-State Conformation Selection

Previous electrophysiological studies showed that deletion of residues 110–136 preserves the principal mechanosensitive gating properties of MscL, although the truncated channel exhibits a higher propensity to occupy subconducting states [21,22]. These findings suggest that the transmembrane core is sufficient to support mechanosensitive gating, whereas the cytoplasmic bundle contributes to the stabilization of conductive conformations and may influence the energetic landscape of gating. We therefore used the same truncated MscL model under all simulation conditions, enabling direct comparison of channel responses within a consistent structural framework.

After completion of the EEF-steered MD simulations for BPTI conformation 6 under an external electric field of 0.10 V/Å, structural clustering analysis was performed to identify representative conformational states sampled throughout the trajectory. All trajectory snapshots were clustered using the MMTSB toolkit with the k-means algorithm and a fixed backbone RMSD cutoff (2.5 Å), grouping structurally similar conformations into distinct clusters based on backbone similarity.

For each cluster, the centroid structure, defined as the frame with the smallest average RMSD relative to all other cluster members, was selected as the representative conformation. These centroid conformations were subsequently evaluated using MM/PBSA free-energy calculations to assess their thermodynamic stability and physical plausibility. Among the resulting conformational states, a representative structure characterized by both a relatively large pore radius and a favorable free-energy profile was selected as the candidate open-state conformation for subsequent pocket identification and ligand docking studies (see below).

This two-step selection procedure, consisting of structural clustering followed by energetic evaluation, ensured that the final open-state model was not only structurally representative of a dominant conformational basin sampled during the simulation, but also energetically stable under the applied field conditions.

### 2.4. MM/PBSA Free-Energy Characterization of BPTI Passing Ec-MscL Channel

To characterize the energy profile experienced by BPTI during translocation through Ec-MscL under an external electric field of 0.10 V/Å, MM/PBSA free energies were calculated for individual frames along the biased MD trajectory and projected onto the reaction coordinate defined by ΔZ, representing the distance between the center of mass of BPTI and the central plane of the pore (Figure 2). The resulting energy distribution revealed distinct energetic basins and barrier regions along the ΔZ coordinate. A relatively deep and broad energy minimum was observed between ΔZ = −25 and −5 Å, suggesting that this region corresponds to a metastable pre-translocation or intermediate translocation state. In contrast, conformations with ΔZ < −35 Å exhibited higher and more variable energy values, indicating a less favorable energetic region near the intracellular entrance of the pore. This observation is consistent with the simulation results obtained under weaker electric fields (e.g., 0.01 V/Å), where BPTI frequently stalled near ΔZ ≈ −35 Å, likely due to insufficient driving force to overcome the associated energy barrier.

To obtain a smoother representation of the underlying energy landscape, ΔZ values were further grouped into discrete 1Å bins, and the average MM/PBSA free energy within each bin was calculated to construct a one-dimensional MM/PBSA energy profile value (Figure 2B). The resulting curve displayed a clear global minimum near ΔZ ≈ −10 Å, which likely represents the most thermodynamically favorable position of BPTI within the pore under the applied biasing conditions.

This free-energy minimum provided a rational basis for selecting candidate open-state conformations. Cluster representative structures located near the profile value minimum (ΔZ ≈ −10 Å), while also exhibiting favorable MM/PBSA energies and high QMEAN6 scores, were prioritized for downstream pocket analysis and virtual screening studies. Together, these results suggest that a stable open-pore conformation can be maintained under EEF bias and that BPTI adopts a metastable intermediate binding position during forced translocation through the Ec-MscL channel.

**Figure 4:**
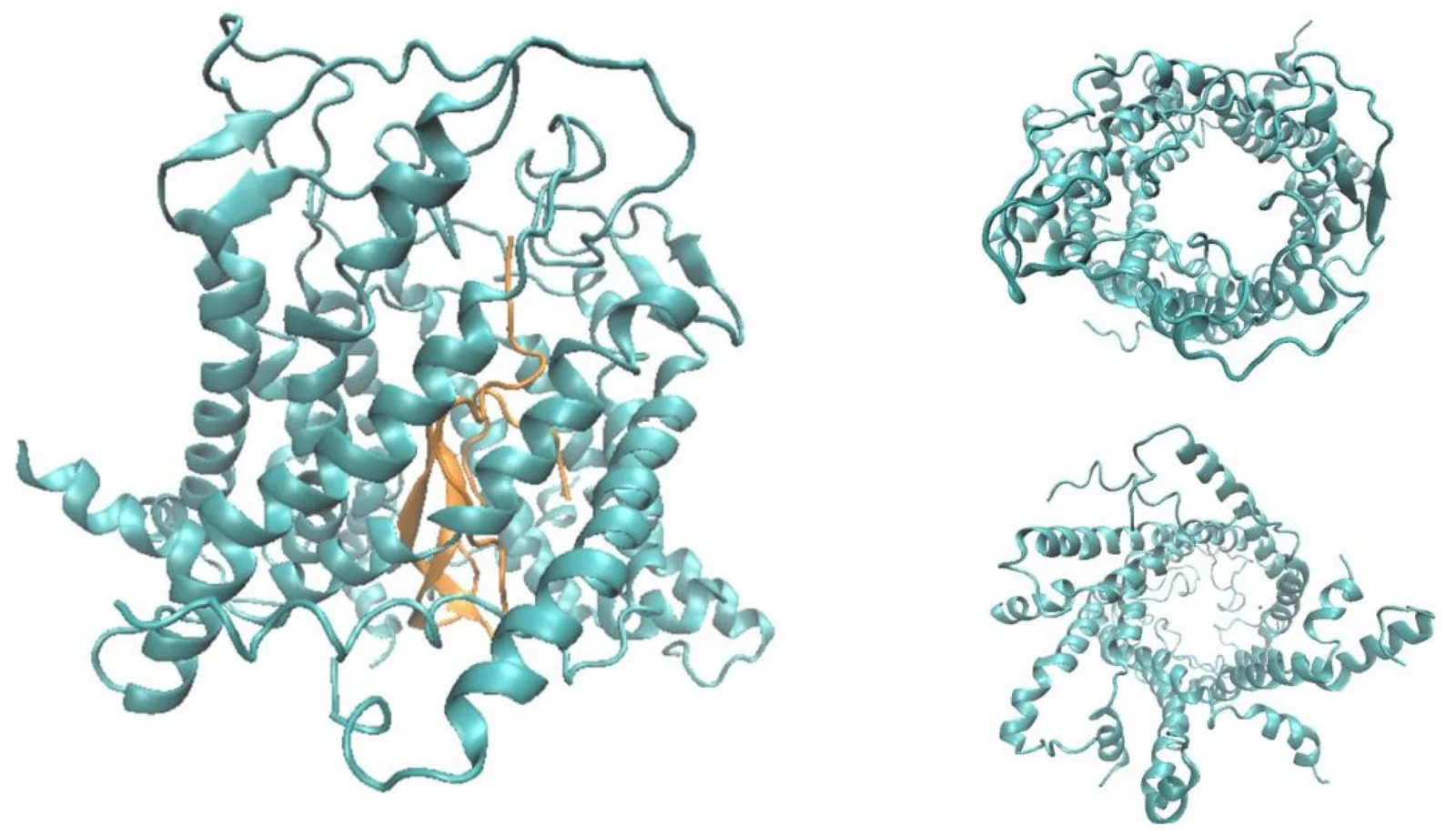
Representative partially open-channel conformation of Ec-MscL at ΔZ ≈ 9.5 Å, corresponding to a metastable intermediate state along the BPTI translocation pathway. The channel protein is shown in cyan, and BPTI peptide is highlighted in orange.

This selection strategy is grounded in thermodynamic principles, where lower-energy conformations generally correspond to higher-probability and more stable states within the conformational ensemble (Figure 4). Collectively, these observations demonstrate that MM/PBSA analysis is not only useful for ranking static conformations but also provides a physically meaningful description of the energy landscape, enabling identification of metastable states and transition regions along the ligand translocation pathway.

### 2.5. Binding Pocket Identification

After selecting the representative open-state Ec-MscL conformation, we applied SiteMap analysis and identified five potential binding pockets, ranked according to SiteScore and DScore (Table 1). The top-ranked pocket, with a SiteScore of 1.174 and a DScore of 1.260, was selected as the docking site for structure-based virtual screening. Glide docking was then performed against the prepared CO-ADD compound library. Based on the docking results, eight top hits with docking score better than −9 kcal/mol were selected for conventional MD simulations to evaluate their ability to stabilize the open state of the Ec-MscL channel. Six independent conventional MD simulations were performed for each ligand-bound MscL system, as well as a ligand-free system for comparison. For each individual simulation, the time length was set to 500 ns, and snapshots were taken every 0.5 ns.

**Figure 5:**
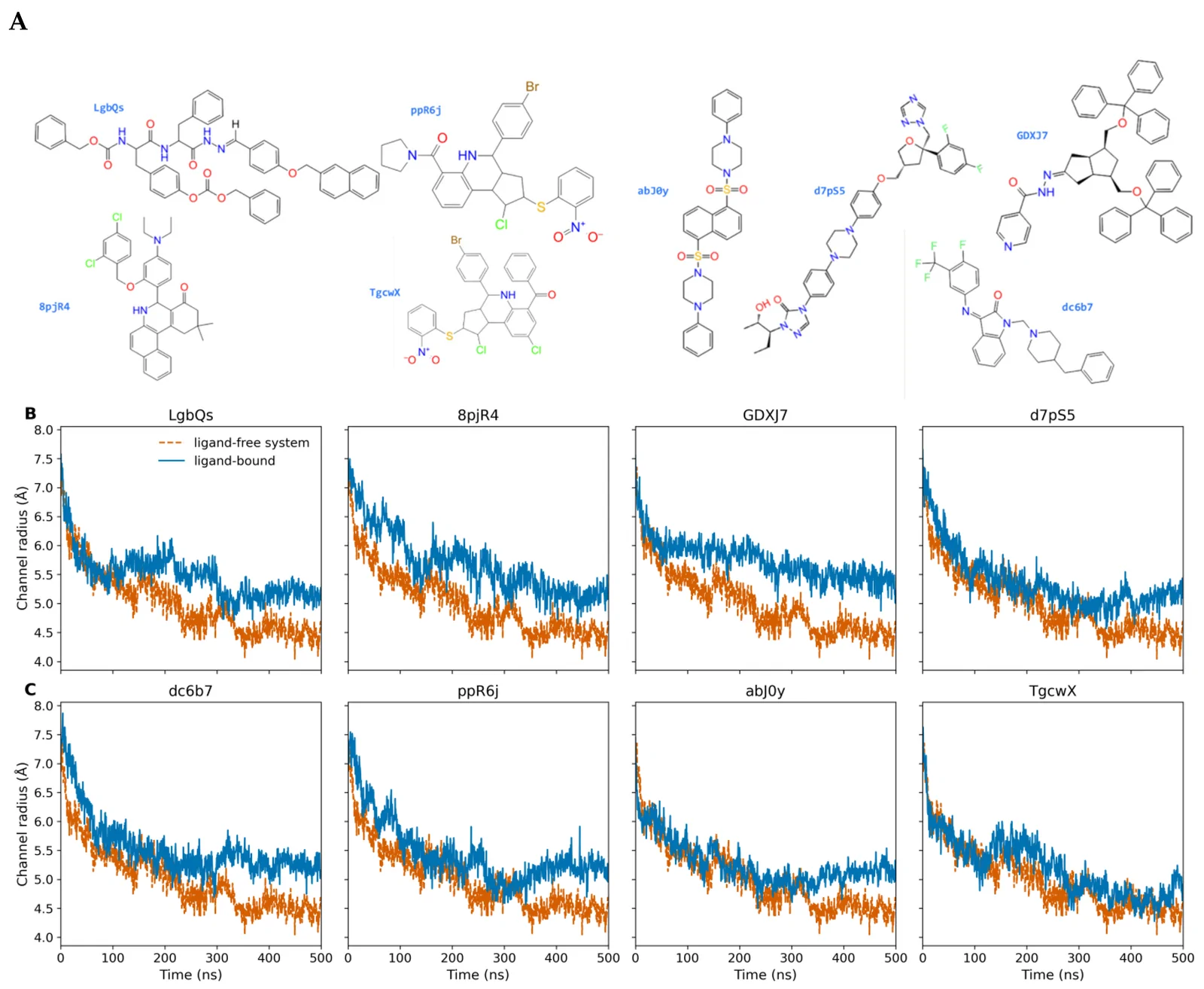
Ligands identified to interfere with Ec-MscL channel gating. (**A**) 2D structures of the eight ligands with the highest docking scores. (**B**) Average Ec-MscL channel radius in the presence of ligands 1–4; (**C**) Time evolution of the Ec-MscL channel radius in the presence of ligands 5–8. The ligand-free control system is shown in orange for comparison. The ligand bound system is colored in blue, with the ligand free system as reference highlighted in orange.

For each snapshot extracted from the six simulation trajectories, the channel radius was calculated, and the averaged values as a function of simulation time are shown in Figure 5. Relative to the ligand-free control system, most ligands slowed or prevented gate closure, thereby maintaining a larger channel radius throughout the simulation. In the control simulation, the channel radius stabilized at approximately 4.6 Å, whereas the top-ranked ligand (LgbQs) maintained an average channel radius of 5.2 Å, indicating sustained pore opening throughout the simulation (Figure 5). Thus, these ligands represent promising candidates for experimental validation as potential antibacterial agents. For the MscL channel radius changes during the simulation, all eight systems exhibit rapid pore-closure phase during the initial 100 ns, followed by a convergence to more stable states. From the perspective of radius, some ligands, such as d7pS5 or abJ0y, displayed a radius decreasing behavior similar to the ligand-free system, and maintained relatively smaller channel radii throughout the simulation. In contrast, ligands such as TgcwX and 8pjR4 sustained comparatively larger average radii. These variations may reflect different ligand-channel interaction modes that modulate MscL gating dynamics.

**Figure 6:**
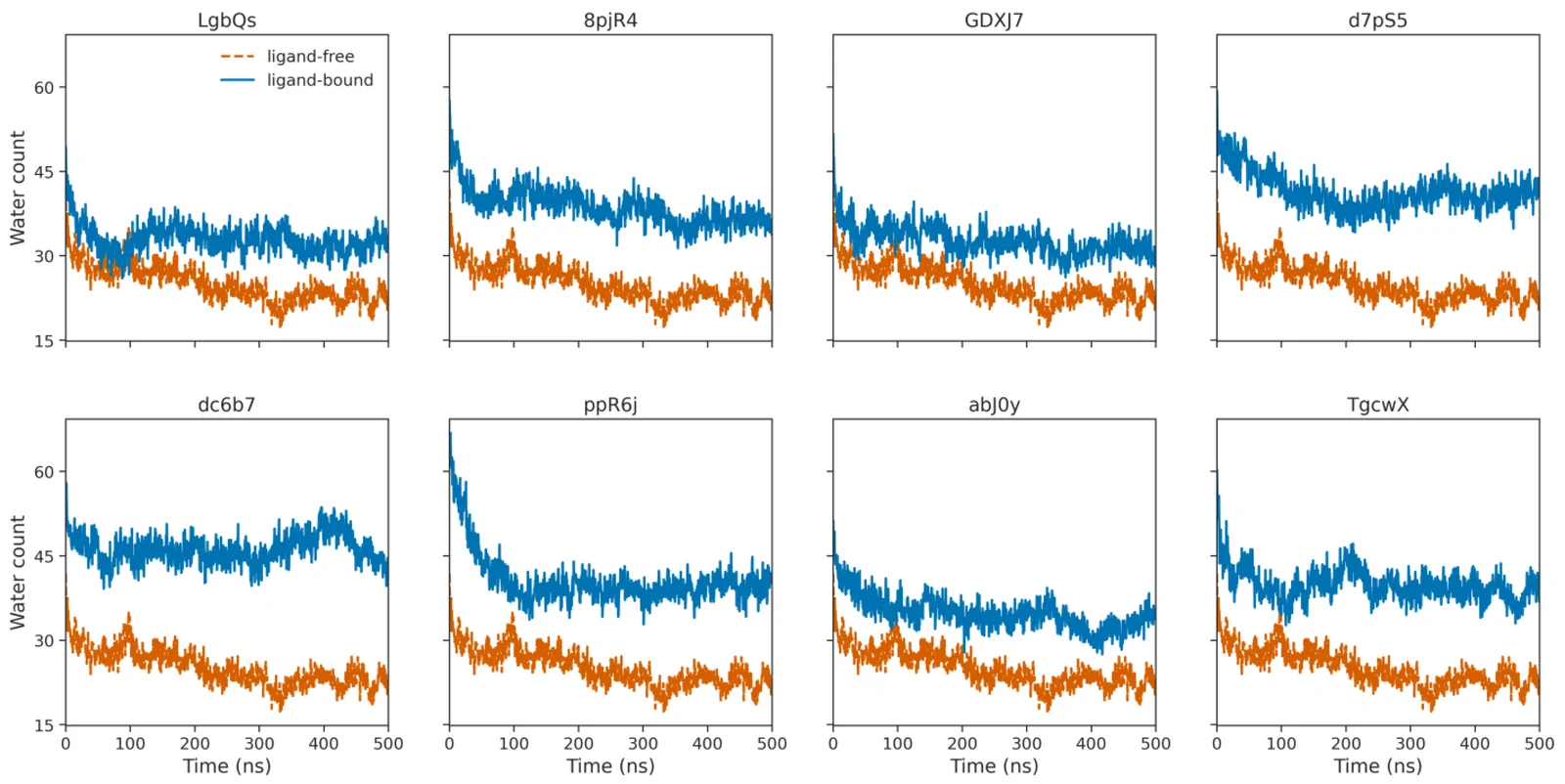
Average number of water molecules within 5 Å of the bottleneck residues V21, V157, V293, V429, and V565 during six parallel 500 ns MD simulations. The orange lines represents the ligand-free Ec-MscL system and serves as the reference. The blue lines correspond to Ec-MscL systems bound to different ligands.

**Figure 7:**
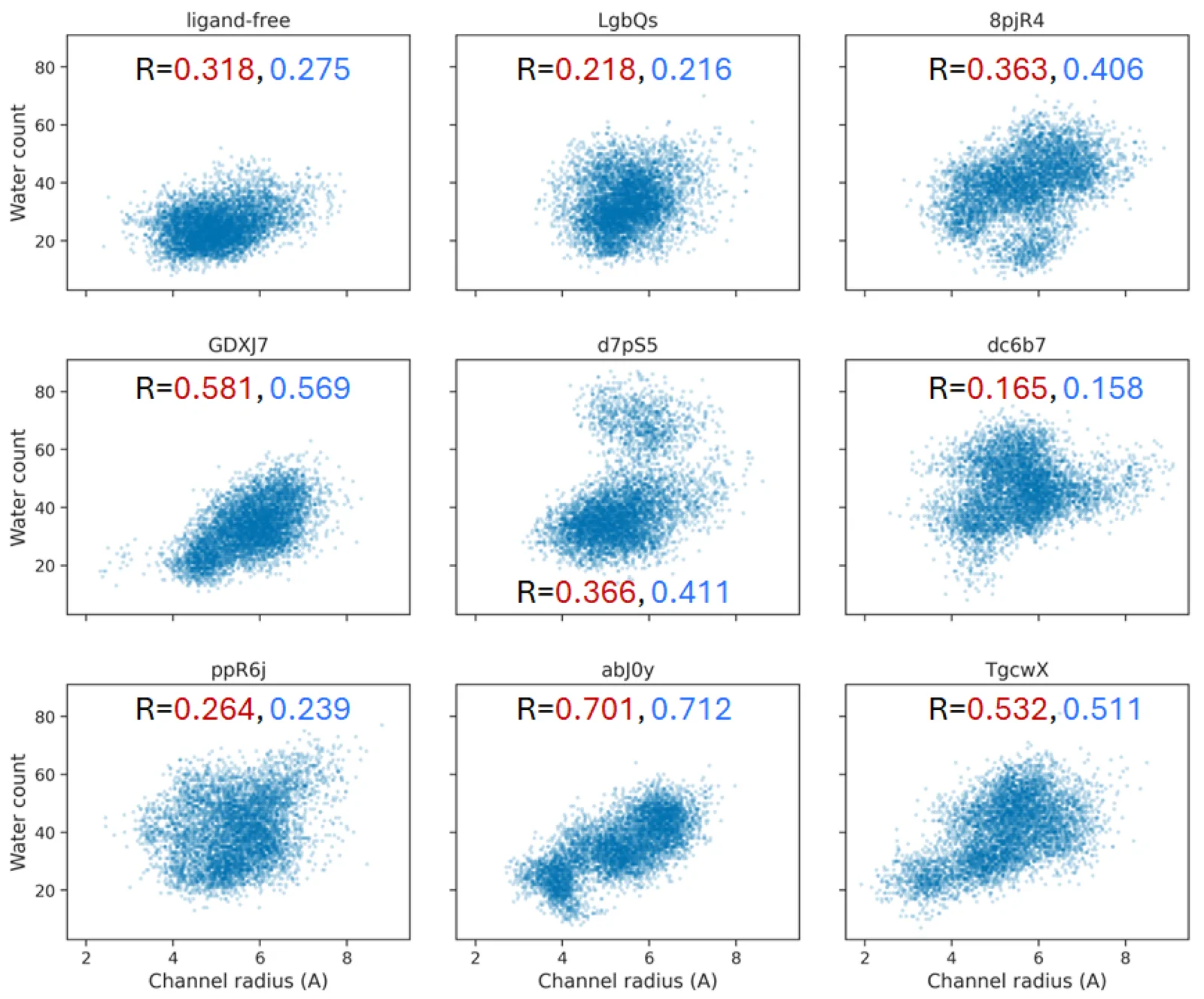
Correlation between channel radius and the number of water molecules located within 5 Å of the bottleneck residues V21, V157, V293, V429, and V565. The data represent averages over all snapshots collected from six independent 500 ns MD trajectories. Pearson (red) and Spearman (blue) correlation coefficients are shown at the top of each panel.

To further evaluate whether maintenance of the pore geometry was accompanied by local wetting near the hydrophobic gate, we calculated the number of water molecules within 5 Å of the bottleneck residues V21, V157, V293, V429, and V565. The water occupancy was averaged over all snapshots collected from six independent simulations (Figure 6). The relationship between channel radius and local water occupancy is shown in Figure 7 and quantified using both Pearson and Spearman correlation coefficients. Ligand-bound systems such as abJ0y and TgcwX exhibited strong positive correlations between water occupancy and channel radius (Pearson R = 0.701 and 0.532, respectively), whereas dc6b7 and LgbQs showed much weaker correlations (Pearson R = 0.165 and 0.218, respectively). Previous computational studies showed that water occupancy in a channel pore depends on both its size and local hydrophobicity [23]. As shown in Figure 5, however, a larger pore radius observed in our simulations did not necessarily correspond to greater water occupancy near the gate. We therefore considered both pore radius and water occupancy as complementary measures to characterize changes in channel structure and local hydration.

### 2.6. In Vitro Experiments Indicate That Identified Compounds Reduce the Viability of MscL-Expressing Cells

**Figure 8:**
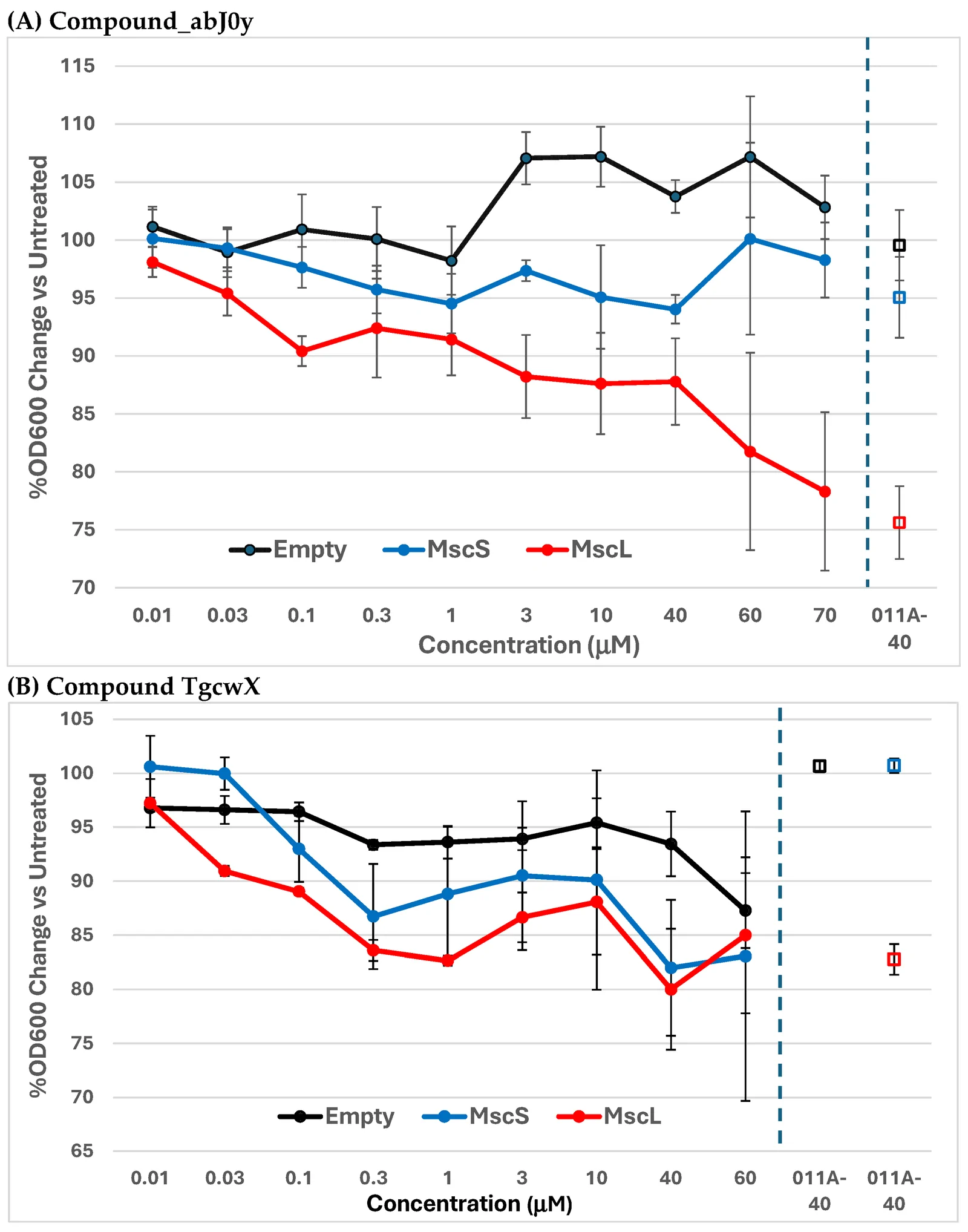
Cell viability of bacterial strain MJF455 (ΔMscL, ΔMscS) carrying vector only (pb10D) in black or expressing either MscS (Blue) or MscL (red) following treatment with inhibitors at different concentrations: (**A**) abJ0y and (**B**) TgcwX. Compound abJ0y exhibits a dose-dependent reduction in viability in MscL-expressing cells. Values are expressed as the percentage decrease in growth in the presence of the compound relative to the untreated samples. *n* = 3 and error bars show standard error of the mean (SEM). The inhibitory activity of 011A, a known MscL inhibitor at 40 μM, is shown on the far right of the plots for comparison. The blue dashed vertical line separates the test-compound data from the 011A positive control.

Four commercially available compounds, d7pS5, TgcwX, LgbQs and abJ0y, were evaluated in the cell viability assays. Among them, abJ0y and TgcwX showed the largest inhibitory effects on the viability of Ec-MscL expressing cells. Figure 8 summarizes the viability profiles of these two compounds across a range of concentrations. In particular, in the MscL-expressing strain, compound abJ0y exhibited a clear concentration-dependent reduction in cell viability, with the most pronounced decrease observed at 70 µM, where mean growth was reduced to approximately 78.3%, compared with 102.8% in the empty plasmid (pB10D) control and 98.3% in the MscS group. At 70 µM, the difference between the MscL group and the empty plasmid (pB10D) control was significant in the unadjusted comparison (*p* = 0.0268), while the difference between the MscL and MscS groups was not significant (*p* = 0.1852). Importantly, no cytotoxicity or nonspecific growth inhibition was detected in the empty vector strain, even at the highest tested concentration. Results are provided in Supplementary Tables S1 and S2.

All tested compounds exhibited poor aqueous solubility and required elevated concentrations of DMSO to remain in solution, which may have limited their apparent activity at micromolar concentrations. Future efforts will therefore focus on structural optimization of these compounds to improve their drug-like properties, including solubility and potency, as well as identification of additional analogs with more favorable pharmacological profiles.

## 3. Methodologies

### 3.1. Molecular Dynamics Simulation System Setup

**Figure 9:**
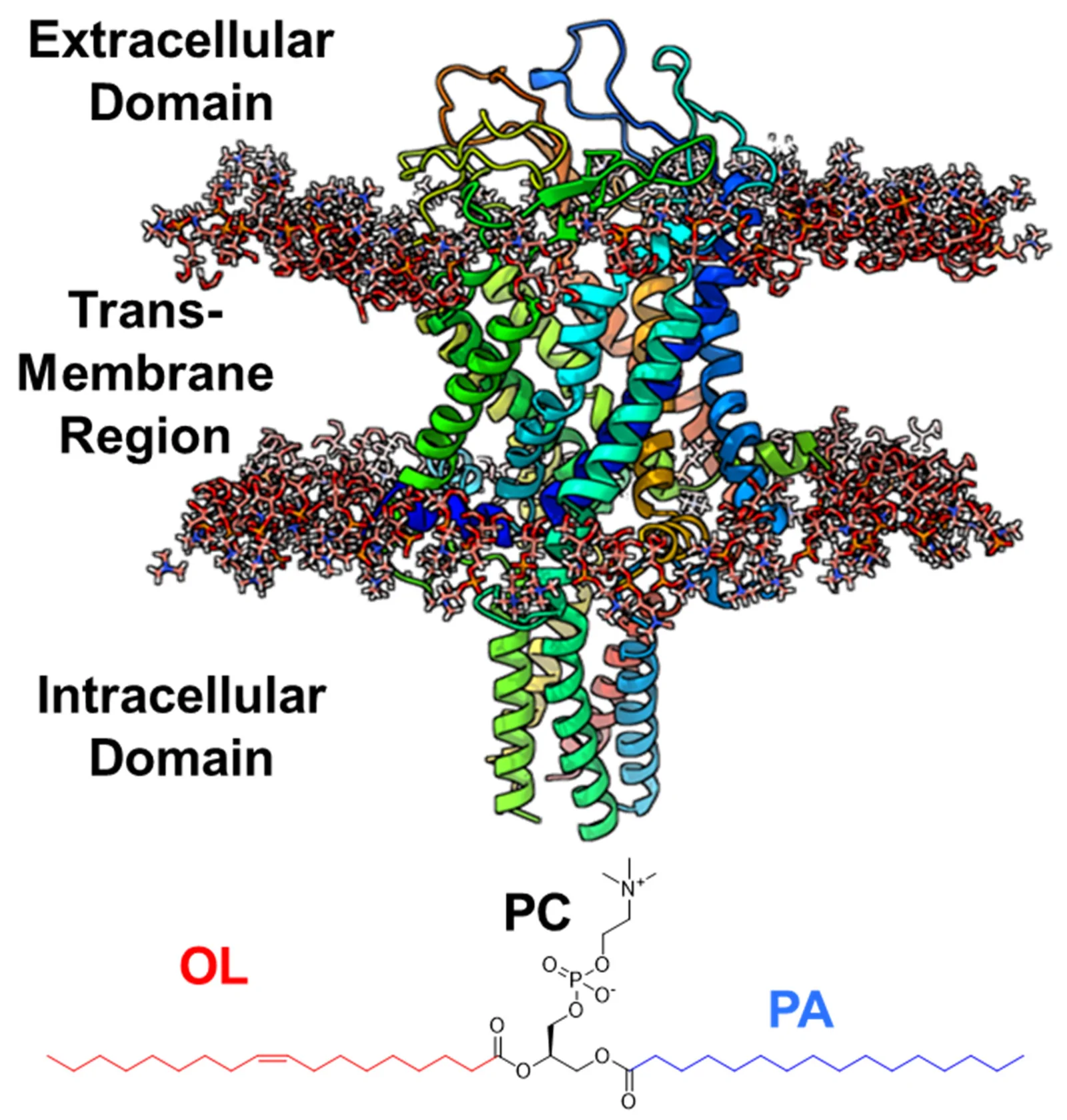
Full structure of the transmembrane channel protein Ec-MscL in its closed conformation embedded in a lipid bilayer composed of 230 POPC molecules. Ec-MscL is represented in cartoon form, while the lipid acyl chains (OL and PA) of POPC are omitted for clarity. Carbonyl carbon atoms of the phosphatidylcholine headgroups are indicated as part of the PC residue.

All molecular dynamics (MD) simulations were based on an Ec-MscL structure, prepared using a protocol consistent with our previous study [18]. The simulation system was constructed using CHARMM-GUI [24] and embedded in a lipid bilayer composed of 230 1-palmitoyl-2-oleoyl-sn-glycero-3-phosphocholine (POPC) molecules (Figure 9). The system was then solvated with 57,441 explicit water molecules and supplemented with 176 K^+^ and 175 Cl^−^ ions to reproduce physiological ionic strength and ensure overall charge neutrality of the simulation box.

Based on prior work, deletion of the C-terminal domain of Ec-MscL was shown to retain the principal gating parameters, although the occupancy of subconducting states was altered [21]. Because the C-terminal cytoplasmic helical bundles are not essential for the principal gating function, we truncated this region to facilitate molecular dynamics simulations of BPTI passage through the channel while retaining the functional transmembrane core. Ec-MscL forms a homopentamer, with each identical monomer comprising 136 residues. Accordingly, residues 1–106 of each monomer were retained, whereas residues 107–136, corresponding to the C-terminal cytoplasmic helical bundle, were removed. The C-terminus of each retained Lys106 residue was modeled as a standard ionized terminal residue in the AMBER topology, including the terminal OXT atom, and the total system charge was neutralized.

The bovine pancreatic trypsin inhibitor (BPTI) is a well-characterized 58-residue globular protein with a molecular weight of approximately 6.5 kDa and is among the smallest and most extensively studied stable protein scaffolds [25,26,27,28]. At physiological pH (7.0), BPTI carries a net positive charge of approximately +6, making it a suitable probe for investigating passage through the Ec-MscL channel under an external electric field [26]. This setup enabled the capture of open-channel conformations along the MD trajectories. Importantly, previous experimental studies using dual-color fluorescence-burst analysis and fluorescence correlation spectroscopy (FCS) have demonstrated that fluorescently labeled BPTI can permeate through the open MscL pore, providing direct experimental support for its translocation capability [5].

**Figure 10:**
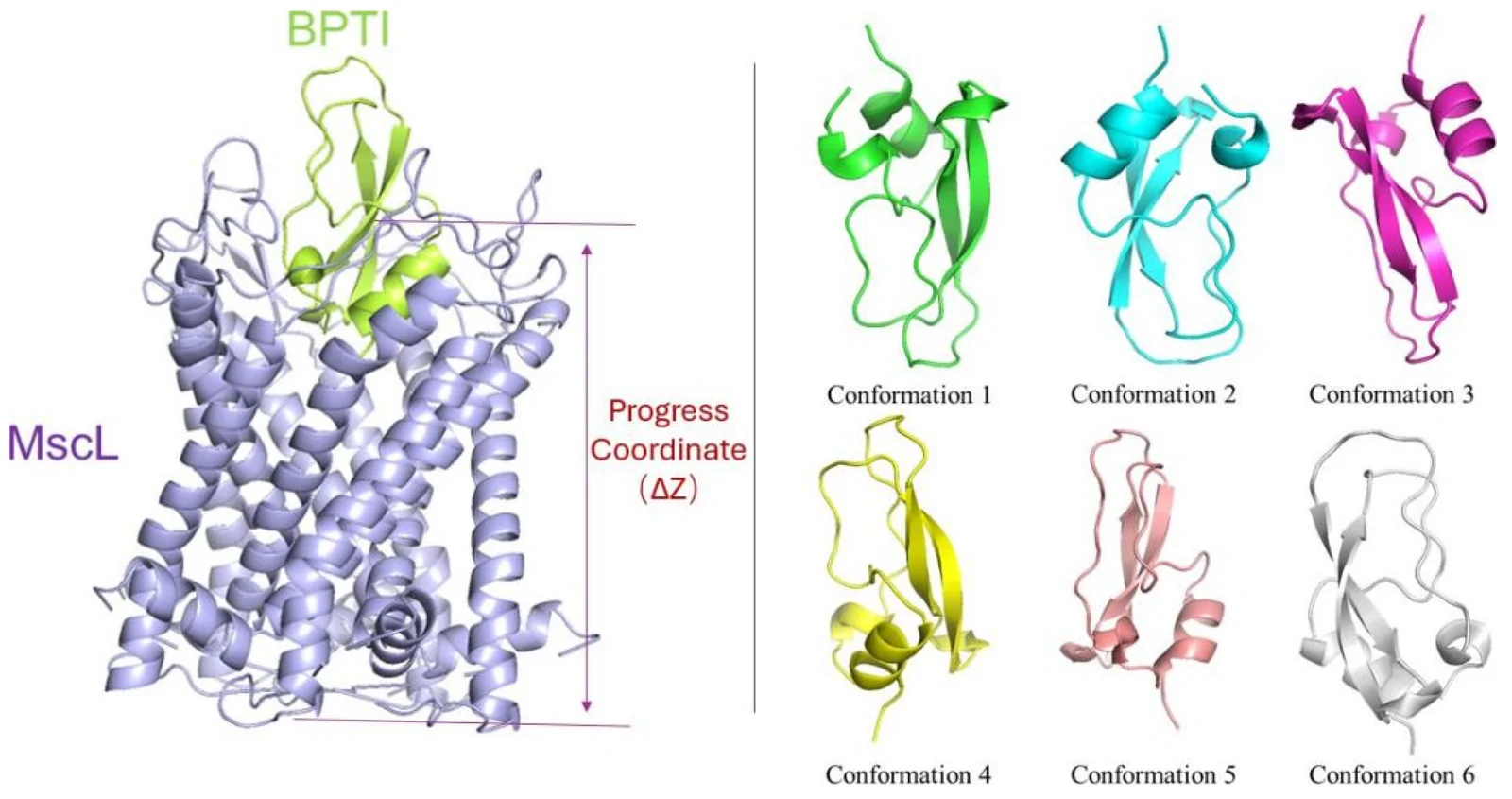
Initial configuration of the Ec-MscL–BPTI complex, with the BPTI peptide positioned at the center of the Ec-MscL pore region. The reaction coordinate (ΔZ) was defined as the distance along the z-axis between the centroid of BPTI and the centroid of five lysine residues (K106, K242, K378, K514, and K650) within the pentameric channel. The six initial conformations of BPTI used in this study are shown on the right side of the figure.

To investigate an open conformation of Ec-MscL while accounting for conformational variability of the protein–peptide system, we generated six initial configurations of the BPTI peptide by sequentially rotating the molecule around the Y-axis in 60° increments within the XZ plane. These conformations were labeled 1–6 (Figure 10). This systematic rotational sampling enabled a more comprehensive exploration of BPTI translocation pathways through Ec-MscL, rather than relying on a single initial orientation. The MD system was described using the ff14SB [29] force field for proteins, lipid14 [30] for the lipid bilayer, and TIP3P [31] for water and ions [32] for ions, respectively.

To quantify the translocation progress of BPTI through the MscL channel, we defined a progress coordinate, denoted as ΔZ, representing the distance along the z-axis between the centroid of the BPTI molecule and the centroid of five lysine residues (K106, K242, K378, K514, and K650) located near the cytoplasmic constriction of the pentameric MscL channel. These lysine residues were previously identified as forming the linkage region between the transmembrane domain and the cytoplasmic bundle region [33]. Monitoring the ΔZ coordinate throughout the simulations enabled precise tracking of BPTI movement relative to the MscL pore.

### 3.2. EEF Molecular Dynamics Simulations

To facilitate translocation of the BPTI peptide through the Ec-MscL channel, we employed a modified version of the AMBER PMEMD simulation package (based on AMBER 16) that enables selective application of an external electric field (EEF) exclusively to the BPTI molecule during both the system preparation and production simulations. The force exerted on one atom by the EEF along the z-direction was defined as:

(1) $$f_{i}^{z}=c_{i}\times {\mathrm{EEF}}^{z}$$

where *c_i_* represents the partial charge of atom *i*, and *EEF^z^* denotes the magnitude of the external electric field applied along the z-axis. The electric field strength was expressed in units of V/Å, with the z-direction oriented perpendicular to the membrane bilayer.

The EEF-driven molecular dynamics (MD) simulation protocol consisted of three major stages: (1) an initial relaxation phase, during which structural constraints were gradually removed to relax the system; (2) an equilibration phase to ensure thermodynamic stability; and (3) a production sampling phase in which BPTI translocation and channel opening events were monitored. Integration timesteps of 1 fs were used during the relaxation and equilibration stages, followed by a 2 fs timestep during the production simulations. Covalent bonds involving hydrogen atoms were constrained using the SHAKE algorithm [34].

The relaxation procedure included a series of 10,000-step energy minimizations designed to remove unfavorable steric contacts and structural strain. During this process, positional restraint force constants were gradually reduced from 20 to 10, 5, 1, and finally 0 kcal/mol/Å^2^ to allow progressive relaxation of the system toward a stable conformational state. Detailed equilibration procedures have been described in our previous study [19].

During the production phase, three external electric fields of 0.10, 0.15, and 0.20 V/Å were applied along the z-axis on BPTI exclusively, to facilitate its translocation and promote channel opening while minimizing structural perturbation to the protein. At each EEF strength, six independent trajectories were performed, each initiated from one of six starting conformations. To further reduce computational cost and accelerate conformational sampling, portions of the cytoplasmic bundle region of Ec-MscL were truncated, as previous studies have demonstrated that the TM1 helices contribute most prominently to the large-scale conformational motions associated with channel gating and opening [35].

### 3.3. Protein Structural Quality Assessment

To evaluate whether EEF-driven sampling introduced progressive deterioration in the structural quality of Ec-MscL, we calculated QMEAN6 Z-scores for snapshots collected along the EEF trajectories [36]. QMEAN6 combines multiple structure-based statistical potentials and sequence–structure agreement terms to assess the compatibility of a protein model, including distance-dependent residue pair interactions, solvation burial terms, local backbone torsion potentials, and agreement between predicted and observed secondary structure and solvent accessibility [37]. These statistical terms are derived from high-resolution experimental protein structures and are used to evaluate the compatibility of a model with stereochemical features commonly observed in native proteins. More negative QMEAN6 Z-scores indicate greater deviations from the structural characteristics represented by the QMEAN6 reference distribution. All QMEAN6 Z-scores were calculated using the Qualitative Model Energy Analysis server (Computational Structural Biology Group, SIB Swiss Institute of Bioinformatics and Biozentrum, University of Basel, Basel, Switzerland), https://swissmodel.expasy.org/qmean (accessed on 10 July 2023). The QMEAN6 Z-score was therefore used to assess the structural plausibility of the EEF-sampled conformations and to evaluate how their structural quality varied across different EEF conditions.

### 3.4. MM/PBSA Free-Energy Calculation and Structure Relaxation

To identify a representative low-energy open-state conformation sampled during EEF-steered MD simulations, we evaluated the free energies of selected snapshots using the Molecular Mechanics Poisson–Boltzmann Surface Area (MM/PBSA) method [38]. For each snapshot, a short 100 ps conventional MD simulation was first performed to relax the structure, followed by MM/PBSA calculations to estimate the total free energy according to Equation (2):

(2) $$\Delta G_{\mathrm{MM}/\mathrm{PBSA}}=\Delta E_{\mathrm{inter}}+\Delta E_{\mathrm{ele}}+\Delta E_{\mathrm{vdw}}+\Delta G_{\mathrm{sol}}^{p}+\Delta G_{\mathrm{sol}}^{\mathrm{np}}-T\Delta S$$

The free energy consists of the internal bonded molecular mechanics energy (Δ*E*_inter_), electrostatic energy (Δ*E*_ele_), van der Waals energy (Δ*E*_vdw_), polar solvation free energy ($\Delta G_{\mathrm{sol}}^{p}$), nonpolar solvation free energy ($\Delta G_{\mathrm{sol}}^{\mathrm{np}}$), and the entropic contribution (*T*Δ*S*). The polar solvation free energy was calculated by solving the Poisson–Boltzmann equation using the Delphi software package (DelPhi 6.2 (f95), April 2014) [39]. The structure with the lowest total free energy was selected as the representative stable open-state conformation for subsequent analyses. Key parameters used in the Poisson–Boltzmann (PB) calculations included interior and exterior dielectric constants of 1 and 80, respectively.

### 3.5. Protein Channel Radius Calculation

Protein channel structures were visualized using PyMOL (version 1.7.4.5), and channel radius calculations were performed using HOLE, a well-established program for analyzing pore dimensions in channel proteins [40]. Owing to the structural complexity of the channel interior, the default parameters for defining the channel direction were not used. Instead, a customized procedure was developed to account for the quasi-symmetry of the Ec-MscL pentamer.

For each structure analyzed, we selected all possible combinations of three out of five glycine residues (G26, G162, G298, G434, and G570), whose *C_α_* atoms correspond to reported bottleneck residues in the channel pore [41]. This resulted in ten unique residue combinations for each structure. For each combination, the centroid of the three selected glycine residues was calculated and used as the CPOINT input in HOLE to ensure that the reference point was located inside the channel pore. The three *C_α_* coordinates define a plane, and the vector perpendicular to this plane was calculated and supplied to HOLE as the channel direction vector. Along each direction vector, the radius of the largest sphere that could be accommodated within the channel was calculated along the pore axis. The maximum channel radius parameter (ENDRAD) was set to 12 Å, and the maximum Monte Carlo simulated annealing step length was set to 0.6 Å. These parameters enabled comprehensive sampling of the pore geometry while maintaining high precision in the radius calculations.

By evaluating ten candidate direction vectors for each channel structure, we obtained a robust estimate of the channel radius while minimizing the influence of local structural variations. Among the ten calculations, the largest finite radius was selected as the final channel radius for the structure. This strategy provides a reliable estimate of the maximum pore dimension along the most favorable channel orientation and therefore serves as an effective measure of the channel’s capacity to accommodate the translocation of molecules such as BPTI.

Channel visualization was performed using a sequential pipeline consisting of sph_process, which converts the HOLE-derived pore-radius profile into a set of connected spheres representing the channel cavity; sos_triangle, which transforms the sphere representation into a smooth triangulated surface mesh of the pore; and Raster3D (version 3.0-7) [42], which generates high-quality three-dimensional renderings of the channel and its internal surface geometry.

### 3.6. Cluster Analysis and Representative Conformation Selection

In addition to radius calculation, following the 100 ps relaxation MD after EEF sampling, all 2404 relaxed conformations sampled during the channel-opening process were clustered to identify representative conformational states. The clustering analysis was performed on all trajectory snapshots using the MMTSB Tool Set (version 1.4, release 2011) [43]. The cluster.pl utility within the MMTSB Tool Set was used to group structurally similar conformations using a k-means clustering algorithm (kclust option). A fixed-radius RMSD criterion, with a radius cutoff of 2.5 Å, was employed to control cluster assignment and ensure that conformations within each cluster remained within a specified structural similarity threshold. This procedure enabled the identification of structurally related conformations and their partitioning into distinct clusters, each corresponding to a local basin in the conformational space explored during the simulations. Within each cluster, the conformation with the smallest average RMSD relative to all other cluster members was selected as the representative structure. Representative conformations exhibiting the most favorable free energies, and therefore likely corresponding to metastable states along the translocation pathway, were selected for subsequent analyses.

### 3.7. Docking Screening

Protein Preparation. The partially open conformation of Ec-MscL, selected based on prior MD simulations, QMEAN assessments, and MM/PBSA energetic evaluation was prepared using the Protein Preparation Wizard in Maestro with default parameters. This workflow included the addition of hydrogen atoms, optimization of hydrogen-bonding networks, and assignment of protonation states for ionizable residues to generate a biologically relevant starting structure for docking studies.

Potential ligand-binding sites within MscL were identified using the SiteMap module implemented in Schrodinger Software Package (Schrödinger Release February 2017) [44]. Binding site evaluation was based on the SiteScore metric, which integrates multiple structural descriptors, including the number of site points (*n*), enclosure (*e*), and hydrophilic character (*p*). The SiteScore is defined as:

(3) $$\mathrm{SiteScore}=0.0733\sqrt{n}+0.6688e-0.20p$$

DScore is a related SiteMap metric developed to assess binding-site druggability using the same general site properties. It was calculated as $0.094\sqrt{n}+0.60e-0.324p$, with the more negative coefficient for the hydrophilic term imposing a greater penalty on highly hydrophilic sites [44].

In general, higher SiteScore values and DScore values indicate more favorable druggable binding pockets. Sites with SiteScore values greater than 1.0 are considered highly promising, whereas values above 0.8 are typically indicative of potential binding pockets suitable for small-molecule docking studies. For DScore measuring druggability, sites with DScore < 0.83, 0.83–0.98, and >0.98 are generally classified as undruggable, difficult, and druggable, respectively.

Ligand Preparation. To identify promising compounds capable of forming stable interactions with Ec-MscL, a ligand library of approximately 100,000 antibiotic-like molecules was curated from the Community for Open Antimicrobial Drug Discovery (CO-ADD), one of the largest open-access antimicrobial screening initiatives [45,46]. The CO-ADD database contains compounds that have been previously synthesized and experimentally evaluated for antimicrobial activity, providing a reliable and readily accessible chemical space for virtual screening and downstream experimental validation.

Three-dimensional ligand structures were generated from 2D simplified molecular input line entry system (SMILES) representations using Open Babel (version 3.1.0; accessed October 2023) under default parameters [47]. Subsequently, ligand preparation was performed using the LigPrep module in Maestro (Schrödinger Release February 2017) [48]. The OPLS3 force field was applied to generate energetically minimized three-dimensional conformations and assign appropriate atomic charges [49]. For each ligand, up to five low-energy stereoisomers were retained to account for stereochemical variability and to improve sampling of potential binding-competent conformations during docking.

Flexible Ligand Docking. The Glide module [50] in Maestro was used to perform all molecular docking calculations in standard precision (SP) mode, providing a balance between computational efficiency and docking accuracy. To define the search space, a 20 Å cubic grid box was centered on each identified binding pocket, ensuring sufficient spatial coverage for ligand translation and rotation within the site. In addition, an inner enclosing box (10 Å in diameter) was applied to focus conformational sampling toward the core region of the binding pocket and improve the efficiency of pose exploration. To bias sampling toward physically and biologically relevant binding modes, intramolecular hydrogen bond formation was treated as a favorable contribution to the docking score, whereas deviations from planarity were penalized as unfavorable contributions.

### 3.8. Conventional Molecular Dynamics Simulations Analyzing Binding Stability

Conventional molecular dynamics (MD) simulations were performed to assess protein–ligand binding stability and to evaluate the effects of antibiotic ligands on the gating behavior of the open Ec-MscL conformation. Based on docking results, the top 15 ligands with the highest docking scores were selected for further investigation. Among these candidates, eight ligands with docking scores better than −9 kcal/mol were selected for conventional MD simulations. For each ligand–receptor complex, six independent 500 ns MD simulations were carried out to capture statistically meaningful effects of ligand binding on the conformational dynamics and pore radius evolution of the Ec-MscL channel.

As a reference, six parallel 500 ns MD simulations were also performed for the ligand-free open Ec-MscL structure. This control set enabled direct comparison of channel behavior in the presence and absence of bound ligands, thereby facilitating identification of ligand-induced deviations in structural dynamics and pore properties. These analyses support subsequent prioritization of candidate hit compounds for experimental binding validation. Channel radius calculations were performed using the HOLE (release 2.2.005, 7 August 2016) [40] with the same parameters described above.

### 3.9. Experimental Validation

To evaluate the biological activity of the selected compounds, bacterial growth assays were performed using the mechanosensitive-channel-deficient *Escherichia coli* MJF455 strain (Frag1, Δ*mscL*::Cm, Δ*yggB*; *yggB* encodes *MscS*), carrying either the empty pB10D vector or pB10D constructs expressing Ec-MscL or Ec-MscS under the control of the lacUV5 promoter. Cultures were grown in CphM containing 100 μg/mL ampicillin to an OD600 of 0.2 and the expression was induced with 1 mM IPTG for 30 min. Cultures had a final OD600 of 0.35 before compound treatment. Cultures were then diluted 1:200 in CphM, and 100 μL was mixed with 100 μL of medium containing the compound at twice its final concentration, giving 200 μL per well.

Cells were exposed to serial dilutions of each compound (1–70 µM) in 96-well plates with three biological replicates per condition. Following overnight incubation at 37 °C, bacterial growth was quantified by measuring the optical density at 600 nm (*OD*_600_), using a Multiskan Ascent 354 plate reader (Thermo Fisher Scientific, Waltham, MA, USA). 011A, the most potent compound identified by us so far, was used as a control compound [51]. More details on in vitro cell-based inhibitory assays were reported elsewhere [32,51].

To improve the solubility of hydrophobic compounds, DMSO was included at a final concentration of 2% in all wells, including control samples. Empty refers to bacteria carrying the empty plasmid (pB10D), while untreated samples contained no test compound but the same 2% DMSO. Blank wells contained the assay medium without bacteria. Blank-corrected *OD*_600_ values were divided by the corresponding untreated values, then averaged across replicates, and results are reported as the mean ± standard error of the mean (SEM). For abJ0y and TgcwX, comparisons with the empty plasmid (pB10D) and MscS controls were performed using two-sided paired *t* tests, with measurements paired by experiment date (*n* = 3). Results are provided in Supplementary Tables S1 and S2, respectively.

## 4. Conclusions

In summary, this study presents an integrated computational-to-experimental framework for the identification and evaluation of small-molecule modulators targeting the *Escherichia coli* mechanosensitive channel (Ec-MscL). Using non-equilibrium molecular dynamics simulations driven by an external electric field (EEF), we captured a metastable open conformation of Ec-MscL induced by peptide-scale permeation of bovine pancreatic trypsin inhibitor (BPTI). This strategy provides a physically interpretable approach for accessing otherwise difficult-to-sample functional states of the channel. The resulting open-state model, evaluated using Molecular Mechanics Poisson–Boltzmann Surface Area (MM/PBSA) free-energy analysis and QMEAN structural quality scoring, provided a structurally plausible balance between channel openness and stability and served as the basis for structure-based virtual screening of antibiotic-like compounds from the Community for Open Antimicrobial Drug Discovery (CO-ADD) database.

Subsequent molecular dynamics simulations identified eight ligands that were predicted to consistently stabilized the metastable open-channel state and impeded channel closure under the simulated conditions. Four representative compounds were selected for preliminary experimental evaluation in MscL-expressing bacterial strains, among which abJ0y exhibited measurable growth-inhibitory effects comparable to those of the known MscL modulator 011A identified in our previous studies. Although quantitative interpretation was partially limited by physicochemical factors such as solubility and optical interference, the observed activity provides experimental support for the predictive utility of the EEF-derived open-state model and the overall structure-enabled screening strategy.

Overall, this study establishes an integrated pipeline spanning EEF-guided conformational sampling, structure-based ligand discovery, molecular dynamics evaluation, and in vitro validation, enabling the rational identification of small-molecule modulators targeting MscL. More broadly, the results demonstrate the potential of non-equilibrium simulation-guided approaches to facilitate drug discovery against mechanosensitive channels and highlight Ec-MscL as a tractable target for antimicrobial modulation.

## Figures and Tables

**Table 1:** SiteScore and DScore values for the top five identified binding pockets of the selected open-state conformation.

Pocket Rank	SiteScore	DScore
1	1.174	1.260
2	1.106	1.209
3	1.026	1.064
4	1.025	1.118
5	1.003	1.066

## Data Availability

All data were generated using publicly available software packages, including AMBER and Glide. The Ec-MscL structures used for the docking studies are available from the authors upon reasonable request.

## Supplementary Materials

The following supporting information can be downloaded at https://www.mdpi.com/article/10.3390/antibiotics15090923/s1. Tables S1 and S2 summarize the bacterial growth assays results for compound abJ0y and Tgcwx, respectively. Videos S1, S2 and S3 are the movies of the first passage of BPTI through the Ec-MscL channel under EEF of 0.1, 0.15 and 0.2 V/Å, respectively.
